# Supplementary material for: Exploring the potential of large language models for integration into an academic statistical consulting service–the EXPOLS study protocol
Source: PLoS One. 2024 Dec 4;19(12):e0308375. doi: 10.1371/journal.pone.0308375 (PMC11616834; doi:10.1371/journal.pone.0308375)
Supplement: S1 File — (PDF) [file pone.0308375.s002.pdf]

Exploring the Potential of Large language models for integration into an academic Statistical Consulting Service - EXPOLS

Principal Investigator

Dr. Susanne Weber
Medical Center – University of Freiburg
Faculty of Medicine
Institute of Medical Biometry and Statistics
Stefan-Meier-Straße 26
79104 Freiburg
E-Mail: susanne.weber@uniklinik-freiburg.de
Tel.: 0761 270-83815

Content

1. Project title, protocol version, date ... 3
2. Synopses ... 4
   2.1 English synopsis ... 4
   2.2 German ... 5
3. Roles and Responsibilities ... 8
4. Funding and conflicting interests ... 9
5. Registration ... 10
6. Background ... 11
   6.1 Statistical consulting ... 12
   6.2 The integration of a LLM into statistical consulting ... 12
7. Research Questions ... 13
8. Primary and Secondary Outcomes ... 14
9. Study Design ... 16
10. Study Population ... 18
11. Instruments ... 20
12. Risk-benefit assessment ... 22
13. Statistical methods ... 23
14. Data management and data privacy protection ... 24
15. Publication plan ... 28
16. Signatures: Principal Investigator(s) / Applicants, Biometrician ... 29
17. References ... 30

Supplement ... 32

Angaben zum Einsatz einer Künstlichen Intelligenz im Forschungsvorhaben ... 32

1. Project title, protocol version, date

Title: Exploring the potential of large language models for integration into an academic statistical consulting service – EXPOLS

Protocol version: v.1.0

Date: 05.06.2024

2. Synopses

2.1 English synopsis

Title	Exploring the Potential of Large language models for integration into an academic Statistical Consulting Service - EXPOLS
Project aims	• to explore the use of Large Language Models (LLMs) in supporting statistical consulting (e.g. by generating research hypotheses, performing (sample size) calculations, developing analysis plans, aiding in reporting statistical findings)<br>• to evaluate the utility, efficiency, communication and satisfaction related to the use of LLMs in statistical consulting from both advisee and consultant perspective<br>• to develop, execute and evaluate a training module for the use of LLMs in statistical consulting<br>• to identify the strengths, limitations, and areas for potential improvement in the use of LLMs for statistical consulting<br>• to explore experiences, attitudes, fears and current practices regarding the use of LLMs of the staff at the Medical Center and the University of Freiburg
Study design	This multimodal study includes four study parts using qualitative and quantitative methods to gather data:<br>• Study part (I): mixed mode study using qualitative and quantitative methods to explore the use of LLMs in supporting statistical consulting and to evaluate the utility, efficiency and satisfaction related to the use of LLMs.<br>○ Semi-structured qualitative interviews to identify relevant patterns from consultant perspective.<br>○ Longitudinal prospective standardised online questionnaire to collect the perspective of consultants.<br>• Study part (II): quantitative standardised online questionnaire to evaluate the training module for consultants and its potential for improvement.<br>• Study part (III): quantitative standardised online questionnaire to evaluate the consulting sessions using LLMs from advisee perspective.<br>• Study part (IV): quantitative standardised online questionnaire to explore experiences, attitudes, fears and current practices regarding the use of LLMs of the staff at the Medical Center and the University of Freiburg.<br>Within this project, ChatGPT, a product of the company OpenAI, represents LLMs as it is used in the university hospital.
Study population (see chapter 10 for details)	• Study part (I): Statistical consultants (pre and post training session)<br>• Study part (II): Statistical consultants (post training session)<br>• Study part (III): Advisees (clinicians and domain experts) associated to the Medical Faculty / Medical Center with varying levels of experience who seek statistical consulting services.<br>• Study part (IV): Members of the Medical Center and the University of Freiburg with varying levels of experience regarding the use of LLMs

Primary Outcome	Assessment of the perceived usefulness, efficiency, and satisfaction of LLM-assisted statistical consulting.
Secondary Outcome	• Compilation of feedback on the benefits and challenges faced by users of LLMs in the consulting process.<br>• Recommendations for the integration of AI tools like LLMs in statistical consulting services.<br>• User-centered development of an informative and sustainable training module.
Time table & working packages	• WP 1: Development of training module: Month 1<br>• WP 2: Execution of training session: Month 2<br>• WP 3: Development of questionnaires, instruments: Month 1<br>• WP 4: Pre-training survey among consultants: Month 1<br>• WP 5: semi-structured qualitative interviews with consultants: Month 2<br>• WP 6: Analysis of qualitative interviews: Month 2,3<br>• WP 7: Post-training survey among consultants (one week/three months/six months after training session): Month 2,5,7<br>• WP 8: Post-consulting survey among advisees: Months 2-7<br>• WP 9: Survey among scientific/ medical staff (members of University/Medical Center): Month 4<br>• WP 10: Analysis and preparation of findings (except follow-up of Study part I,III): Month 5<br>• WP 11: Dissemination of results, data and evaluation of the need for further research (Study part II, IV): Month 6<br>• WP 12: Analysis and preparation of findings for follow-up (Study part I, III): Month 7

2.2 German

Titel	Erkundung des Potenzials von Large Language Models bei der Integration in einen akademischen statistischen Beratungsdienst - EXPOLS
Projektziele	• Die Nutzung von Large Language Models (LLMs) zur Unterstützung der statistischen Beratung erforschen (z.B. durch Generierung von Forschungshypothesen, Durchführung von (Stichprobengrößen-)Berechnungen, Entwicklung von Analyseplänen, Unterstützung bei der Berichterstattung statistischer Ergebnisse)<br>• Den Nutzen, die Effizienz, die Kommunikation und die Zufriedenheit im Zusammenhang mit der Nutzung von LLMs in der statistischen Beratung aus der Perspektive von Ratsuchenden und Beratenden evaluieren<br>• Ein Trainingsmodul für die Nutzung von LLMs in der statistischen Beratung entwickeln, durchführen und bewerten<br>• Stärken, Grenzen und Bereiche für potenzielle Verbesserungen im Einsatz von LLMs für die statistische Beratung identifizieren<br>• Erfahrungen, Einstellungen, Ängste und aktuelle Praktiken im Umgang mit LLMs des Personals am Universitätsklinikum und der Universität Freiburg erforschen

Studiendesign	Diese multimodale Studie umfasst vier Studienteile, die qualitative und quantitative Methoden zur Datenerhebung nutzen:<br>• Studienteil (I): Mixed-Mode-Studie unter Verwendung qualitativer und quantitativer Methoden zur Erforschung der Nutzung von LLMs zur Unterstützung der statistischen Beratung und zur Bewertung des Nutzens, der Effizienz und der Zufriedenheit im Zusammenhang mit der Nutzung von LLMs.<br>o Semi-strukturierte qualitative Interviews zur Identifizierung relevanter Muster aus der Beraterperspektive.<br>o Longitudinale standardisierte Online-Umfrage zur Erfassung der Perspektive von Berater*innen.<br>• Studienteil (II): Quantitative, standardisierte Online-Umfrage zur Bewertung des Trainingsmoduls für Berater*innen und dessen Verbesserungspotenzial.<br>• Studienteil (III): Quantitative, standardisierte Online-Umfrage zur Bewertung der Beratungssitzungen unter Verwendung von LLMs aus der Perspektive der Ratsuchenden.<br>• Studienteil (IV): Quantitative, standardisierte Online-Umfrage zur Erforschung von Erfahrungen, Einstellungen, Ängsten und aktuellen Praktiken im Umgang mit LLMs des Personals am Universitätsklinikum und der Universität Freiburg.<br>In diesem Projekt repräsentiert ChatGPT, ein Produkt des Unternehmens OpenAI, die LLMs, da es im Universitätsklinikum verwendet wird.
Studienpopulation (siehe Kapitel 10 für Details)	• Studienteil (I): Statistische Berater*innen (vor und nach der Schulung)<br>• Studienteil (II): Statistische Berater*innen (nach der Schulung)<br>• Studienteil (III): Ratsuchende (Kliniker*innen und Fachexpert*innen), die der Medizinischen Fakultät/dem Medizinischen Zentrum angehören und über unterschiedliche Erfahrungsstufen verfügen und die statistische Beratung in Anspruch nehmen.<br>• Studienteil (IV): Alle Mitglieder des Universitätsklinikums und der Universität Freiburg mit unterschiedlichen Erfahrungsniveaus im Umgang mit LLMs
Primäre Zielgrößen	Bewertung der wahrgenommenen Nützlichkeit, Effizienz und Zufriedenheit von LLM-unterstützter statistischer Beratung.
Sekundäre Zielgrößen	• Bestandsaufnahme von Feedback über die Vorteile und Herausforderungen, die Nutzende von LLMs im Beratungsprozess erleben.<br>• Empfehlungen für die Integration von KI-Tools, wie LLMs, in statistische Beratungsdienste.<br>• Nutzerzentrierte Entwicklung eines informativen und nachhaltigen Trainingsmoduls.
Zeitplan & Arbeitspakete	• WP 1: Entwicklung des Trainings: Monat 1<br>• WP 2: Durchführung des Trainings: Monat 2<br>• WP 3: Entwicklung von Fragebögen, Instrumenten: Monat 1<br>• WP 4: Vor-Training-Umfrage unter Berater*innen: Monat 1

	• WP 5: Teilstrukturierte qualitative Interviews mit Berater*innen: Monat 2<br>• WP 6: Analyse der qualitativen Interviews: Monat 2,3<br>• WP 7: Nach-Training-Umfrage unter Berater*innen (eine Woche / drei Monate / sechs Monate nach dem Training): Monat 2,5,7<br>• WP 8: Nach-Beratungs-Umfrage unter Ratsuchenden: Monate 2-7<br>• WP 9: Umfrage unter wissenschaftlichem/medizinischem Personal (Mitglieder der Universität / des Medizinischen Zentrums): Monat 4<br>• WP 10: Analyse und Vorbereitung der Ergebnisse (ohne Follow-Up für Studienteil I,III): Monat 5<br>• WP 11: Verbreitung der Ergebnisse, Daten und Bewertung des Bedarfs an weiterer Forschung (Studienteil II, IV): Monat 6<br>• WP 12: Analyse und Vorbereitung der Ergebnisse für den Follow-Up (Studienteil I, III): Monat 7

3. Roles and Responsibilities

Principal investigator:

Dr. Susanne Weber, Institute of Medical Biometry and Statistics, Faculty of Medicine and Medical Center – University of Freiburg, Germany, Susanne.Weber@uniklinik-freiburg.de, Tel. 0761 270-83815

Co-Principal investigator:

Urs A. Fichtner, Institute of Medical Biometry and Statistics, Section Healthcare Research and Rehabilitation Research, Faculty of Medicine and Medical Center – University of Freiburg, Germany, Urs.Fichtner@uniklinik-freiburg.de, Tel. 0761 270-36420

Participating scientists:

Prof. Dr. Harald Binder, Institute of Medical Biometry and Statistics, Faculty of Medicine and Medical Center – University of Freiburg, Germany, harald.binder@uniklinik-freiburg.de, Tel. 0761 270-83744

Dr. Erika Graf, Institute of Medical Biometry and Statistics, Faculty of Medicine and Medical Center – University of Freiburg, Germany, Erika.Graf@uniklinik-freiburg.de, Tel. 0761 270-837431

Jochen Knaus, Institute of Medical Biometry and Statistics, Faculty of Medicine and Medical Center – University of Freiburg, Germany, Jochen.Knaus@uniklinik-freiburg.de, Tel. 0761-270-83739

Georg Koch, Institute of Medical Biometry and Statistics, Faculty of Medicine and Medical Center – University of Freiburg, Germany, Georg.Koch@uniklinik-freiburg.de, Tel. 0761 270-83851

Dr. Jörg Sahlmann, Institute of Medical Biometry and Statistics, Faculty of Medicine and Medical Center – University of Freiburg, Germany, Joerg.Sahlmann@uniklinik-freiburg.de, Tel. 0761 270-83748

Dominikus Stelzer, Institute of Medical Biometry and Statistics, Faculty of Medicine and Medical Center – University of Freiburg, Germany, Dominikus.Stelzer@uniklinik-freiburg.de, Tel. 0761 270-83852

Prof. Dr. Martin Wolkewitz, Institute of Medical Biometry and Statistics, Faculty of Medicine and Medical Center – University of Freiburg, Germany, Martin.Wolkewitz@uniklinik-freiburg.de, Tel. 0761 270-83810

Authors' contributions: SW and MW initiated the study design. UF, SW and JK drafted the trial protocol. UF conceived the questionnaires. JK is responsible for data privacy and research data management. SW and UF will conduct the statistical and qualitative analysis. All authors contributed to refinement of the study protocol and approved the final version.

4. Funding and conflicting interests

This study is financed by internal funds. The participating scientists declare no conflicting interests. Mostly all involved researchers are trained statistical consultants.

5. Registration

This study is registered at the Freiburg Registry of Clinical Studies (https://frks.uniklinik-freiburg.de) under the ID: FRKS004971

6. Background

The progress of Artificial Intelligence (AI), especially in the area of Large Language Models (LLMs) is currently accelerating at an ever-faster pace (1). LLMs are artificial neural networks trained by using self- and semi-supervised learning. Popular examples are OpenAI's GPT models, Google's PaLM, DeepMinds Gemini and Meta's LLaMA and Microsoft's KOSMOS (2).

LLMs become more and more attractive in various contexts of the scientific process, e.g. supporting the production of scientific texts, knowledge synthesis, translation – but also data analysis. In the medical context, the use of LLMs has especially been examined in order to support the communication with the patients, (3–5). Not only in the context of medical research, the integration of LLMs is a cutting-edge advancement that is revolutionizing research across various fields. (6) examined the use of AI at a higher education landscape via a survey among students in Germany. LLMs can take over "roles", e.g. those of programmers or data analysists using statistical software, e.g. Python or R and can answer to questions that deal with problems of data analysis. It is notable, that LLMs have the capability to perform tasks beyond their original intended purposes. (7) investigated how access to ChatGPT 4 impacts productivity and quality of consultants within a global management consulting company. LLM tools can act as "statistical consultant" supporting to find answers and solutions for programming issues and bug fixing. Furthermore, a new plugin, called "Advanced Data Analysis" (ADA) was released for ChatGPT 4 that provides specific features for data evaluation. After an update, this feature is now available as GPT called “Data Analyst”. With this feature, users can upload their data and let the system analyse them by using textual questions (called “prompts”). ADA introduces an extensive range of new application areas. For instance, (8) investigated opportunities and limitations of this plugin for hydrological analysis. However, both the use of LLMs as "consultant", as well as "data analyst" face several risks (9,10). First, the quality of the outcomes of LLMs has to be verified and might depend on several aspects, as e.g. the quality of training data, the difficulty of the question and the wording of the prompts. Second, the quality of the outcomes needs to be validated and verified and it stands to question whether the outcome quality can be judged in a general manner. As long as the outcome quality and precision of the operations of the LLM is not guaranteed, it might produce errors of different levels of severity. Third, without deeper understanding and education, the advice-seeking person might not be able to correctly differentiate between invalid and proper answers or analysis results. Fourth, uploading data onto a server can be problematic for reasons of data protection and limits the use of ADA.

At the Medical Faculty of the University of Freiburg, statistical consulting is offered to every member of the faculty who searches supervision or support of questions related to data analysis. In some exceptional cases, even the whole analysis process is executed by the statisticians employed there. Since many medical researchers have limited education in data science, this consultation is offered to support good scientific practice using adequate analysis methods by well-trained field experts. Depending on the statistical expertise of the advisees and the complexity of the analysis questions, the consultation offer varies between a unique e-mail-based advice over two or three face-to-face or web-conference consulting sessions to possibly even programming meetings in presence of the advisee. In order to sustain high quality in statistical consulting, a standardized training and orientation concept has been developed for general quality improvement. This concept has been mandatory to be absolved for consultant beginners since 2019. Additionally, regular bi-weekly meetings are held for collegial exchange, where consultation cases are presented for joint discussion of consulting practices.

It is foreseeable that LLMs will play a role in the future handling of data analysis and thus, in the procedures that are aligned to statistical consultation. Both advice-seeking scientists and statistical field experts might make use of such tools to support their work. The question of how to integrate

LLMs in research is, however, not limited to the medical field. LLMs can be used for text and code production and thus, shows potential for all research areas, including teaching. (11) addressed opportunities, threats, and strategies of ChatGPT for education and research. However, many contextual factors might play a role in order to determine a qualified use of LLMs with high efficiency, utility and user satisfaction. Those factors might lie in the persons interacting with the systems, e.g. level of education in statistics, competence and experience in using LLMs, attitudes and fears towards LLMs and personal preferences. Additionally, also factors within the technology used might play an important role, e.g. the outcome quality of the LLM, its interoperability, its flexibility, its transparency, the repeatability of the outcomes as well as its comparability and ease of use.

6.1 Statistical consulting

Many statistical research organizations offer methodological support to clinical and life science researchers. This may happen in individual consultations without further involvement, in project-specific co-operations or in long-term collaborations among domain experts and biostatisticians (12). Consultation can be provided on aspects of study design and methodological evaluation, and the advice given can refer to basic research or to clinical research projects.

At the Institute of Medical Biometry and Statistics (IMBI), a free consulting service is operated by a cross-section of all employed statisticians and mathematicians, with more than 20 IMBI scientists providing consulting in addition to their research activities. Over 250 requests for advice are processed each year. Those seeking advice are members of the Medical Faculty of the University of Freiburg or the Freiburg University Medical Center. The advisees range from students with questions about doctoral projects and postdocs to experienced research group leaders and principal investigators. The contents addressed range from study planning including sample size calculation and planning of the statistical analysis in a study protocol through application of statistical methodology in a given data set.

At the IMBI, and hence in EXPOLS, consulting services are defined as follows:

> "Advising in a single or a few advising session(s) (sometimes called consulting service), where the advisee poses questions on statistical aspects in a particular project and the adviser discusses possible solutions but does not further engage in the project." (12)

In particular, no further engagement means that no data analysis is performed by the consultant.

As there is a wide range in the research areas, research questions and statistical expertise of advisee there is a huge heterogeneity between the consulting sessions.

6.2 The integration of a LLM into statistical consulting

At the Institute of Medical Biometry and Statistics, each statistical consultant has received access to ChatGPT 4 and is advised to integrate the LLM in the consultations. Due to the considerable heterogeneity among consulting sessions, the tasks for which the LLM can be used will also vary. Potential uses of the LLM include pre- or post-session preparation, real-time assistance, data analysis guidance, drafting documentation, serving as an educational tool, and many more. One aim of this project is to assess how this integration works and how it can support statistical consulting.

7. Research Questions

Within this project, we aim to answer the following research questions:

1. How can LLMs be used to support statistical consulting?

    1.1 How do advisees and consultants perceive and rate the use of LLMs in statistical consulting?
    1.2 What are the strengths, limitations and areas of improvement in the use of LLMs for statistical consulting?
    1.3 What factors play a role in determining the use of LLMs with high efficiency, utility and user satisfaction?

2. Is the developed training module for statistical consultants and (future) scientists efficient and sustainable?

3. What are the experiences, attitudes, fears, beliefs and current practices regarding the use of LLMs?

8. Primary and Secondary Outcomes

This study is divided into four sub-studies using different methods to gather data. Each sub-study addresses one or more research questions and thus has its own measurement goals und study population (see section Study population). However, as this is an exploratory study, no hypotheses will be formulated in advance and thus, no clearly prioritized outcome measures can be derived. The outcome measures are displayed by study part in subsequent Table 1:

Study part	Population	Outcomes	Research question
I: Mixed mode study using qualitative and quantitative methods (online, standardised and online, semi-structured)	Statistical consultants (Institute of Medical Biometry and Statistics)	Quantitative:<br>Result expectation<br>Result achievement<br>Satisfaction with use of LLM<br>Quality Assessment<br>Communication Assessment<br>Efficiency Assessment<br>Future potential<br>Perceived risks and limitations<br>Qualitative:<br>Result expectation<br>Perceived barriers<br>Perceived potential<br>Possible pitfalls and solutions<br>Experiences	1, 1.1, 1.2, 1.3
II: Quantitative standardised online questionnaire	Statistical consultants (Institute of Medical Biometry and Statistics)	Appropriateness of the time frame of the training module<br>Structure of the content of the course<br>Perceived value of content<br>Perceived learning success<br>Quality in communication<br>Motivation of the course<br>Preparation quality of the course<br>Willingness to recommend the course<br>Use of training session for future consulting tasks<br>Strengths and limitations, improvement potential<br>Overall quality rating of course	2
III: Quantitative standardised online questionnaire	Advisees (Clinicians and domain experts)	Evaluation of the absolved consulting session (captured via regular evaluation form as a score and categorized)<br>Satisfaction with the use of LLM in consultation<br>Quality assessment<br>Communication assessment<br>Efficiency assessment	1.1
IV: Quantitative standardized online questionnaire	Scientific employees (researchers and clinician scientists at the University Medical Center	Attitudes and beliefs towards the use of LLM<br>Experiences with the use of LLM<br>Current usage of LLM and contexts<br>Perceived benefits in using LLM<br>Perceived barriers in using LLM	3

	and the Albert-Ludwigs-University Freiburg)	Knowledge of or participation in development of guidelines / rules towards the use of LLM<br>Changes in practice in line with the introduction of LLM<br>Differences across faculties and research fields	

Table 1: Study Outcomes

9. Study Design

This monocentric study will utilize qualitative and quantitative methods to explore the use of LLM in an academic statistical consulting service from both consultant and advice-searching staff. Each of the four sub-studies addresses different research questions and is organized in working packages (WP) (see Table 2).

Working package (WP)	Month						
	I	II	III	IV	V	VI	VII
WP 1: Development of training - course conceptualization	■						
WP 2: Training session with all statistical consultants		■					
WP 3: Development of questionnaires, instruments, interview guideline	■						
WP 4: Pre-training survey among consenting consultants	■						
WP 5: Semi-structured qualitative interviews with consultants		■					
WP 6: Analysis of qualitative interviews and synthesis of results		■	■				
WP 7: Post-training survey among consultants (T1: one week after training, T2:three months, T3: six months)		■			■		■
WP 8: Continuous post-consulting survey among advisees		■	■	■	■	■	■
WP 9: Survey among all members of the Medical Faculty / Medical Center				■			
WP 10: Analysis and preparation of findings, knowledge synthesis					■		
WP 11: Dissemination of results, data and evaluation of need for further research (Study part II, IV)						■	
WP 12: Analysis and preparation of findings for follow-up (Study part I, III)							■

Table 2: Working packages and time table

WP 1: To develop the training module, existing teaching material from online resources, prior course visits and own preparatory works will be consolidated. The course consists of a lecture part and a hands-on part. The whole teaching session is estimated to take approximately 2-3 hours. Sustainable reuse potential of the course will guide the development of the training module.

WP 2: The course will be offered in a synchronous hybrid format to enable participants to take part from home office. We offer up to three different dates to ensure that every statistical consultant can take part in the training session. Participation will be mandatory as part of consultants' work-related duties. The course will be offered to all consultants employed at the Institute of Medical Biometry and Statistics and is not restricted to those who participate in the study. The course will be conducted by Susanne Weber, the principle investigator of this project.

WP 3: For all study parts, we will make use of standardized online questionnaires implemented via REDCap. Those questionnaires will be constructed based on existing literature and earlier used items and scales in other, comparable contexts. The questionnaires will be offered in bilingual form (GER, ENG). The interview guideline applied in study part I will be developed within a consortium of field-experts from Sociology, Empirical Social Science, Statistics, Health Services Research, Medicine and Mathematics.

WP 4: Before the training session, we will ask consenting statistics consultants to fill out a short questionnaire provided via REDcap on their expectations about the use of LLM in statistical consulting. Furthermore, we will collect data on potential predictors that might have influence on the adaptation and use of LLM in statistical consulting.

WP 5: With a purposive sampling approach, we will select consultants and will conduct semi-structured guideline-based qualitative interviews to gather in-depth insights into their perceptions regarding the use of LLM in statistical consulting, the associated barriers, limitations and potential benefits. The interviews will be conducted via bilateral Webex-Meetings. The interviews will be digitally recorded.

WP 6: All interviews of WP 5 will be transcribed verbatim and analyzed qualitatively by SW and UF following a parallel coding approach according to Kuckartz (13). The results will be used to refine survey instruments that will be used in WP 7 and 9.

WP 7: After all consultants participated in the training session, they are asked to actively involve LLM in their subsequent consultation sessions. At t1 (one week after training session), t2 (two months after training session) and t3 (5 months after after training session), the consultants will receive a standardized online questionnaire provided via REDcap.

WP 8: All advisees participating in the study will be asked to fill out an evaluation questionnaire after their consultation session. This questionnaire is attached as a link to the regular evaluation of the counselling and is executed as a separate REDCap form. The questionnaire also captures whether or not a LLM was used in the consulting session and what the reasons for non-use were. The survey will also contain questions regarding the advisee's perception towards the use of LLM during the session.

WP 9: Scientific employees at the University Medical Center and the Albert-Ludwig-University Freiburg will be asked to complete a standardized online questionnaire provided via REDCap on a voluntary basis. For recruiting, we will use several different methods to ensure reaching all staff involved in research. The questionnaire will be online for 14 days.

WP 10: The baseline data of Study parts I to IV will be analyzed exploratory and in a descriptive manner. Potential correlations or group-differences will be tested bivariately.

WP 11: The quantitative data and the results will be published in adequate scientific journals (see publication strategy) and needs for further research will be evaluated.

WP 12: The follow-up data of Study parts I and III will be analyzed exploratory and in a descriptive manner. Results will be published in adequate scientific journals.

A detailed overview of the study recruitment workflow is illustrated in Section 14 Data management and data privacy protection.

10. Study Population

The study population varies and thus is described for each study part.

Study part I (Mixed mode study using qualitative and quantitative methods (online, standardised, semi-structured)):

Inclusion criteria: All potential study participants must be members of the statistical consulting team at the time of data collection and have signed a declaration of informed consent. The statistical consulting team consists of 24 consultants. Assuming a participation rate of 80-90% the estimated sample size for the quantitative part is n=20; estimated sample size for the qualitative part: n=6.

Due to the interest already expressed among the consultants, the assumed participation rate seems realistic. We expect a high level of intrinsic motivation based on professional involvement. Participation can take place during regular working hours so that no additional private time resources need to be invested. In order to ensure a high participation rate we implement repeated reminders.

Study part II (Quantitative standardised online questionnaire):

Inclusion criteria: All potential study participants must be members of the statistical consulting team at the time point of data collection and have given electronic informed consent. Similar to study part I, the estimated sample size is n=20.

Study part III (Quantitative standardised online questionnaire):

Inclusion criteria: All potential study participants must have absolved at least one statistical consulting between the date of the teaching of the consultants and the end of the observation period. Electronic informed consent needs to be obtained in advance. There are at least 15 consultations per month. With a data collection period of 6 months this results in at least 90 consultations. Assuming a participation rate of 33% the estimated sample size is n=30.

Study part IV (Quantitative standardized online questionnaire):

Inclusion criteria: All potential study participants must be employed as scientific or medical staff at the University of Freiburg or the University Medical Center or must work closely with science. The declaration of consent is integrated into the start of the online survey. The source population consists of approximately 5.800 scientific employees. In addition, employees working closely to science, but not officially declared as scientists, are also invited to participate. Assuming a response rate of 10-20% the estimated sample size is 600-1200.

Exclusion criteria:

All members that do not fulfil the inclusion criteria will be excluded. Furthermore, potential participants will be excluded if they do not have sufficient language skills in either German or English.

Recruitment:

Study participants for study part I and II are employed in our institute and will be contacted via their associated company e-mail address. Project members are part of the study population and can participate both in the qualitative and the quantitative part. The principal investigators have no authority to issue directives within the consulting team. There are no conflicts of interest, as participation in the study is voluntary and no supervising dependencies are apparent. Participation or

non-participation in the study has no impact on the employment. To ensure this, the questionnaires are treated confidential.

For the qualitative study, a purposive sampling approach will be chosen and participants will be recruited by personal contact.

Study participants for study part III will be recruited via the statistical consultant.

Study participants for study part IV will be recruited via multiple media, e.g. newsletters, as well as newsfeeds, e.g. of the University Medical Center.

11. Instruments

The following data / instruments will be collected / used:

Study Part	Data / Instrument
I	Experience as consultant<br>Sex<br>Typical consultation format<br>References used for consultation<br>Experience with use of AI for statistics<br>Satisfaction with use of AI<br>Technology Readiness (TRI 2.0; (14))<br>- Optimism (4 items)<br>- Innovation (4 items)<br>- Discomfort (4 items)<br>- Insecurity (4 items)<br>Result expectation (15)<br>- Performance Outcome Expectation (POE) (3 Items)<br>- Self-Evaluative Outcome Expectation (SEOE) (3 Items)<br>- Social Outcome Expectation (SE) (3 Items)<br>Perceived benefits and disadvantages in using AI<br>Usage of ChatGPT after training session<br>Reasons for non-usage
II	Learning success<br>Competence gain<br>General teaching competence<br>Potential use of ChatGPT<br>Preferred teaching mode<br>Overall rating<br>Recommendation potential: Net Promoter Score (16)
III	Use of AI during consultation<br>Satisfaction with use of AI during consultation<br>Usability of AI (UMUX, (17))<br>Perceived benefits and disadvantages in using AI
IV	Status group<br>Experience in science<br>Faculty membership<br>Sex<br>Experience using AI: areas<br>Potential use of AI in the future: areas<br>Difficulties using AI<br>Technology Readiness (TRI 2.0; Parasuraman 2014)<br>- Optimism (4 items)<br>- Innovation (4 items)<br>- Discomfort (4 items)<br>- Insecurity (4 items)<br>Result expectation (Niederhauser & Perkmen 2010)<br>- Performance Outcome Expectation (POE) (3 Items)<br>- Self-Evaluative Outcome Expectation (SEOE) (3 Items)<br>- Social Outcome Expectation (SE) (3 Items)<br>Experience and potential for training sessions with the topic AI

	Experience in using AI for teaching<br>Fears about the role of AI in the future<br>Knowledge, use and need for guidelines regarding AI<br>Use of AI in the patient-provider relationship (only medical staff)

Table 3: Instruments used for EXPOLS

12. Risk-benefit assessment

Individual benefit associated with study participation

Study part I, II: The study participants are trained in the use of LLMs and can therefore develop a differentiated opinion on integrating modern AI tools for their work.

Study part III: Study participants learn about potential benefits and risks for their own methodological data analysis under expert guidance.

Study part IV: There is no individual benefit to participating in the anonymous survey.

Burdens and risks associated with study participation

Study part I, II: Familiarization with new tools is always necessary for scientific staff. The additional workload is minimal due to potentially longer counselling sessions.

Study part III: there is a risk of potential minimal increased time effort in statistical consultation.

Study part IV: no risk.

Termination criteria

None required.

Statement on medical justifiability

None required.

13. Statistical methods

Sample size calculation: Since this study is of explorative nature and we aim to conduct a survey with the full basic population, sample size calculation is not applicable.

1. Study part I, II: Statisticians (consultants): The sample size planning is based on the current consultants (N=24). Assuming a participation rate of 80-90%, we expect data of 20 statisticians.
2. Study part III: Domain experts (advisees): Considering January until May 2023 as reference over 15 consultations per month can be expected. With a duration of 6 months for data collection of this subpopulation and an expected response rate of 33%, we expect data of n=30 domain experts (advisees).
3. Study part IV: scientific employees: 2,623 scientists were employed 2022 at the University and 3,189 persons were employed in 2022 with association to the Medical Faculty working in science. In addition, employees working closely to science, but are not officially declared as scientists, are also invited to participate. Assuming a response rate of 10-20% the estimated sample size is 600-1,200.

Methods: Analyses will be explorative.

1. Qualitative Analysis:
   Qualitative data from Study I will be transcribed verbatim and then will be analyzed with the structuring content analysis approach (13).
   Free-text responses from Study II-IV will be thematically analyzed to identify common themes and impressions.
2. Quantitative Analysis:
   Data collected through Likert-scale or multiple-choice questions will be described using descriptive statistics. Absolute frequencies will be provided for the number of consultations and consultants. The number of evaluations will be presented for both consultations and consultants (absolute and relative). Ratings of consultations through the evaluation form will be described using arithmetic mean with a 95% confidence interval, standard deviation, minimum, 25th percentile, median, 75th percentile, maximum, and the count of complete and missing values.
   For the pre-post comparison of ratings, the Mann-Whitney U test will be employed.

14. Data management and data privacy protection

Detailed information and procedures are described in the attached data protection concept.

14.1 Study recruitment workflow

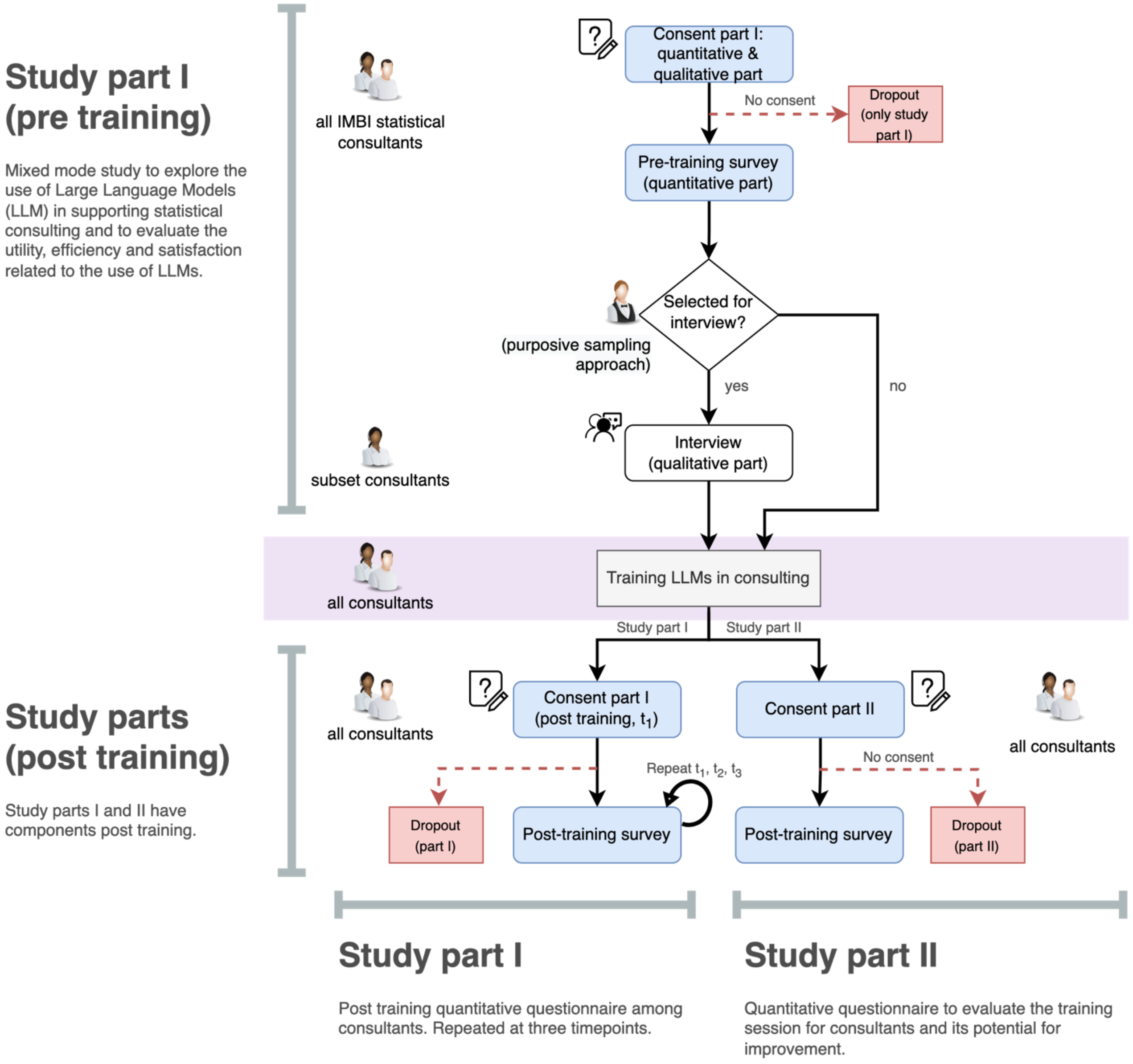

Figure 1 - Recruitment scheme study parts I & II

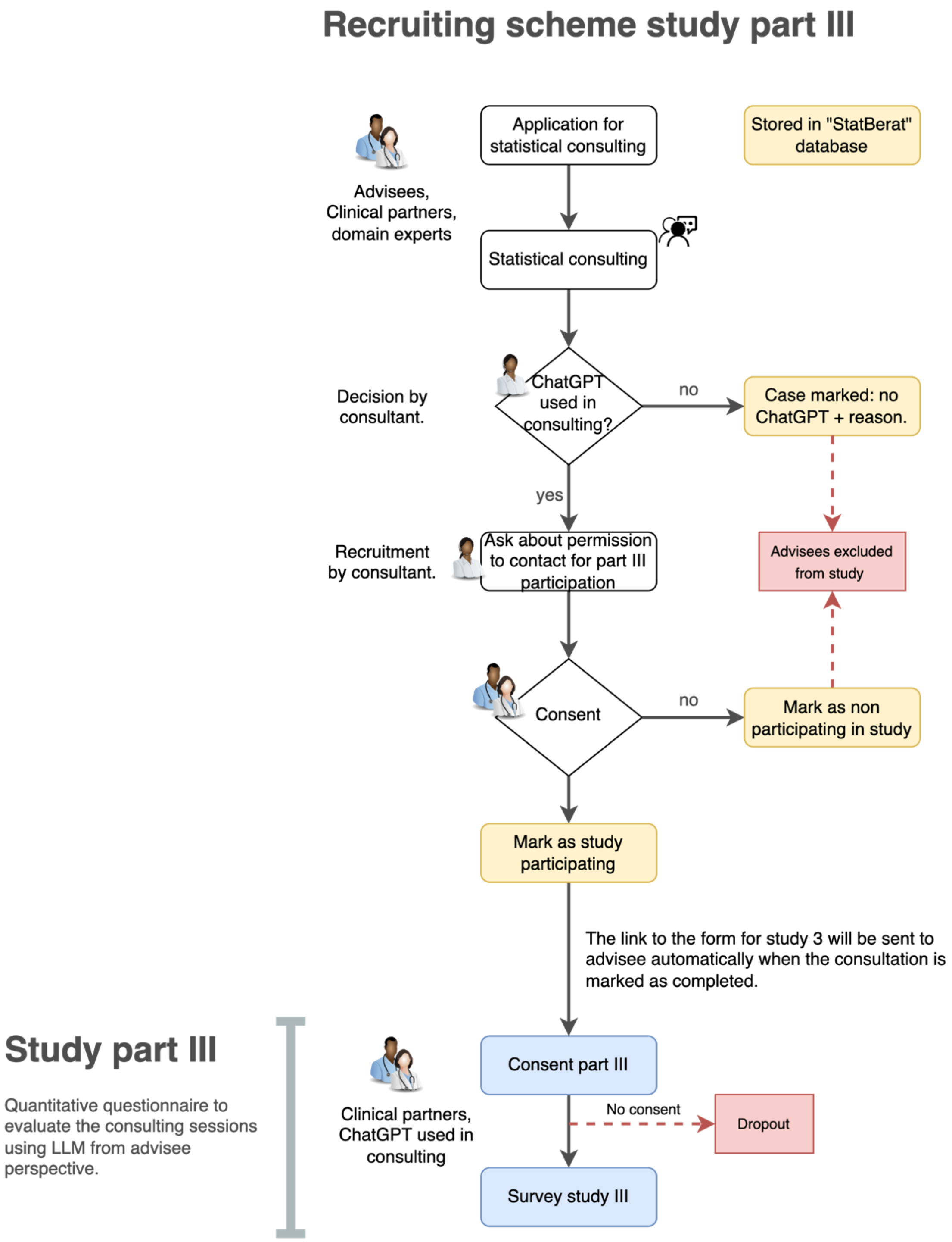

Figure 2 - Recruitment scheme part III

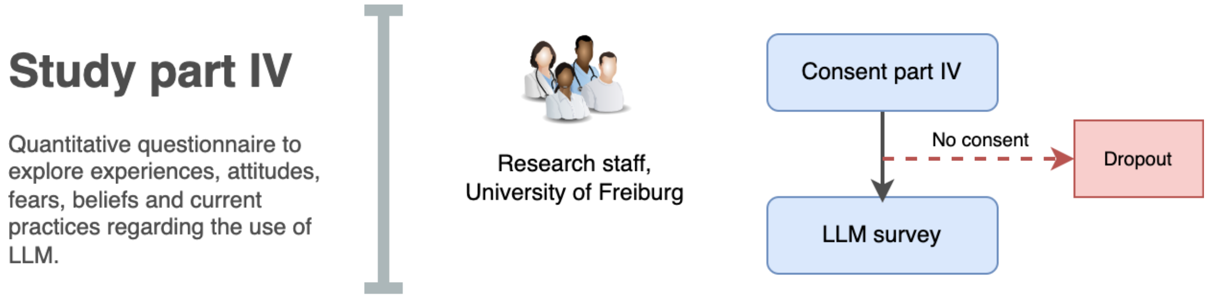

Figure 3 - Recruitment scheme part IV

General: Data in statistical consulting and use of the "ChatGPT" service (OpenAI)

As a matter of principle, the consultants do not accept transmission of any research data as part of the statistical counselling at the IMBI; for the majority of cases the activity is purely consultative. However, the consultants are free to enter into a co-operation project with the advisees if they are interested in methodological issues or for other scientific reasons. For data protection reasons, those cooperations are not covered by the general regulations of the statistical consulting and therefore must be evaluated and documented separately as a normal scientific project.

This means that the data on which the consulting activities are based are not available at the IMBI and are not transferred to the Institute.

In the context of the present study, a large language model (LLM) supports the consulting activities and is subject to the restrictions of statistical consulting, so no data is transferred to the IMBI, stored or processed at the IMBI. There are certain LLMs that can be used in local data centers (e.g. Lama), but these do not yet offer the range of functions of ChatGPT and the Advanced Data Analyses (ADA) function in particular can currently only be used here. The ChatGPT language model used in the study can only be used as a commercial service from a non-European company (OpenAI) in an American data center. The establishment of third-country processing with the conclusion of an order data processing contract with correspondingly extended standard contractual clauses is not intended. This means that the transfer of data relevant to data protection law, i.e. generally clinical study or routine data, to the ChatGPT web service is not permitted within the scope of this study.

The creation of simulation data, which may be transferred for the use of ADA, should be generated under the guidance of the consultant – if ChatGPT is used for simulation data creation, no original patient data must be used. All statisticians are obliged to comply with the standards of the University Medical Center and try to sensitize the advisees so that data is not imported into ChatGPT or other services under data protection regulations outside the counselling process.

Survey data (quantitative data)

Detailed information and procedures are described in the attached data protection concept.

Study part I-IV: The questionnaires are collected via the REDCap data capture report form system. It is provided by the Clinical Trials Unit (ZKS Freiburg). Technically, the public instance is used, which allows the questionnaires to be used outside the University Medical Center network (essential for parts III and IV with potential participants from pre-clinical institutes and the University).

Analysis data sets (cleaned questionnaires) are transferred as CSV files to a network drive of the University Medical Center. Access is restricted to the study evaluators.

Study part I, II: Due to the small number of cases and the known participants (statistical advisory team of the IMBI), there is an increased risk of combinatorial re-identification of using the quantitative data. Here, specific information such as "years of professional experience" is already sufficient for re-identification. This means that the quantitative data sets are excluded from publication and the questionnaire is designed in such a way that it can be used without any consequential risk.

Survey data – pseudonymization/anonymization:

Study part I: participants are pseudonymized to allow for contact for interviews.

Study part II: participants are anonymous in the survey (not in the qualitative part).

Study part III: Surveys are pseudonymized in order to combine the study response with the evaluation response of the overall consultation. Only study leaders have access to the pseudonymization list.

Study part IV: participants are anonymous in the survey.

Free text fields are cleaned before evaluation: any potentially identifying information is stripped and information is categorized where specific terms could lead to additional risk of re-identification. For details, please consult the data privacy concept.

Consent

Electronic consents are obtained for all parts of a multi-part study. For part III, verbal consent is given for contact during consultations, and questionnaire-related consents are strictly managed, with applications to statistical consulting anonymized one year post-consultation and retained for two years post-study. Details for handling and consent, please consult the data privacy concept.

Audio files

Study part I: Interviews are recorded for later transcription. This transcription is done during the study period. The interview data is stored on a University Medical Center network drive and is only accessible to the study evaluators.

Data publication

In line with good scientific practice, the data is published as far as possible.

Study part I-IV: all free text fields are cleared and categorized. Sensible information in free text will be removed at all.

Study part I, II: no data will be published.

Study part III: study data is published, but without the connected information on the statistical consulting case.

Study part IV: study data will be published for reuse of the scientific community.

15. Publication plan

All data will be processed and analyzed anonymously. The preliminary publication plan is listed below (Table 4).

Title / subject	First author	Co-Authors
The EXPOLS study: The study protocol	UF	SW, EG, DS, JS, GK, JK, MW, HB
Development and evaluation of an LLM training for statistical consulting	SW	UF, EG, DS, JS, GK, JK, MW, HB
Perspectives regarding LLM use in statistical consulting – a qualitative study	UF	SW, EG, DS, JS, GK, JK, MW, HB
Implementation of LLM in statistical consulting – exploratory study	SW	UF, EG, DS, JS, GK, JK, MW, HB
Data Article: Data of the EXPOLS Study	UF	EG, DS, JS, GK, JK, MW, HB, SW
Thoughts, fears, experiences, attitudes and practices towards the use of LLM in the statistical and clinical field – exploratory study	UF, JK	SW, EG, DS, GK, JS, JK, MW, HB

Table 4: Publication plan

16. Signatures: Principal Investigator(s) / Applicants, Biometrician

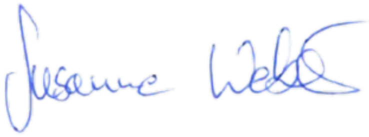

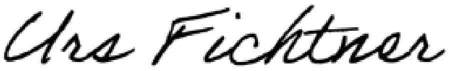

17. References

1. De Angelis L, Baglivo F, Arzilli G, Privitera GP, Ferragina P, Tozzi AE, et al. ChatGPT and the rise of large language models: the new AI-driven infodemic threat in public health. Front Public Health. 2023 Apr 25;11:1166120.

2. Naveed H, Khan AU, Qiu S, Saqib M, Anwar S, Usman M, et al. A Comprehensive Overview of Large Language Models. 2023 [cited 2023 Dec 13]; Available from: https://arxiv.org/abs/2307.06435

3. Montastruc F, Storck W, de Canecaude C, Victor L, Li J, Cesbron C, et al. Will artificial intelligence chatbots replace clinical pharmacologists? An exploratory study in clinical practice. Eur J Clin Pharmacol. 2023 Oct;79(10):1375–84.

4. Witzsch UKF, Borkowetz A, Enzmann T, Rodler S, Leyh-Bannurah SR, Loch T, et al. Digitalisierung in der Urologie – Herausforderung und Chance. Urol Heidelb Ger. 2023;62(9):913–28.

5. Ayers JW, Poliak A, Dredze M, Leas EC, Zhu Z, Kelley JB, et al. Comparing Physician and Artificial Intelligence Chatbot Responses to Patient Questions Posted to a Public Social Media Forum. JAMA Intern Med. 2023 Jun 1;183(6):589.

6. Garrel J, Mayer J, Mühlfeld M. Künstliche Intelligenz im Studium Eine quantitative Befragung von Studierenden zur Nutzung von ChatGPT & Co. 2023;84.

7. Dell'Acqua F, McFowland E, Mollick ER, Lifshitz-Assaf H, Kellogg K, Rajendran S, et al. Navigating the Jagged Technological Frontier: Field Experimental Evidence of the Effects of AI on Knowledge Worker Productivity and Quality. SSRN Electron J [Internet]. 2023 [cited 2024 Jan 5]; Available from: https://www.ssrn.com/abstract=4573321

8. Irvine DJ, Halloran LJS, Brunner P. Opportunities and limitations of the CHATGPT Advanced Data Analysis plugin for hydrological analyses. Hydrol Process. 2023 Oct;37(10):e15015.

9. Yu P, Xu H, Hu X, Deng C. Leveraging Generative AI and Large Language Models: A Comprehensive Roadmap for Healthcare Integration. Healthc Basel Switz. 2023;11(20).

10. Guo Z, Jin R, Liu C, Huang Y, Shi D, Supryadi, et al. Evaluating Large Language Models: A Comprehensive Survey. 2023 [cited 2023 Dec 13]; Available from: https://arxiv.org/abs/2310.19736

11. Rahman MdM, Watanobe Y. ChatGPT for Education and Research: Opportunities, Threats, and Strategies. Appl Sci. 2023 May 8;13(9):5783.

12. LeBlanc M, Rueegg CS, Bekiroğlu N, Esterhuizen TM, Fagerland MW, Falk RS, et al. Statistical advising: Professional development opportunities for the biostatistician. Stat Med. 2022 Feb 28;41(5):847–59.

13. Kuckartz U. Qualitative Inhaltsanalyse: Methoden, Praxis, Computerunterstützung. 4. Auflage. Weinheim Basel: Beltz Juventa; 2018. 240 p. (Grundlagentexte Methoden).

14. Parasuraman A, Colby CL. An Updated and Streamlined Technology Readiness Index: TRI 2.0. J Serv Res. 2015 Feb;18(1):59–74.

15. Niederhauser DS, Perkmen S. Beyond self-efficacy: Measuring pre-service teachers' Instructional Technology Outcome Expectations. Comput Hum Behav. 2010 May;26(3):436–42.

16. Mandal PC. Net promoter score: a conceptual analysis. Int J Manag Concepts Philos. 2014;8(4):209.

17. Finstad K. The Usability Metric for User Experience. Interact Comput. 2010 Sep;22(5):323–7.

Supplement

Angaben zum Einsatz einer Künstlichen Intelligenz im Forschungsvorhaben

In unserem geplanten Projekt EXPOLS wird ChatGPT 4 zum Einsatz kommen. Weiterführende Informationen können dem Studienprotokoll entnommen werden.

1. Zweck und ggfs. Namen der KI oder der Algorithmen.
   In diesem Forschungsvorhaben geht es darum, den Einsatz von einem Large Language Model, ChatGPT, in der Nutzung für statistische Beratungen zu untersuchen.

2. Wird die KI im Rahmen dieses Projektes erstmals entwickelt oder weiterentwickelt, oder handelt es sich um den Einsatz eines fremd-entwickelten KI-Systems und / oder proprietäre Software, mit deren Hilfe Fragestellungen des Projektes beantwortet werden sollen?
   Eingesetzt wird das kommerzielle geschlossene System ChatGPT 4.

3. Wo und von wem wird die Hardware und das KI-System verwaltet (gehostet)?
   Das Hosting erfolgt beim einzigen Anbieter des KI-Modell, dem amerikanischen Anbieter OpenAI. Entsprechend wird eine amerikanische Cloud genutzt.

4. Wo und in welcher Form werden die Projektdaten gespeichert und verarbeitet?
   Im KI-Modell werden keine personenbezogenen oder schützenswerten Daten in ChatGPT verarbeitet.

   Projektdaten werden ausschließlich auf Datenspeichern der Universitätsklinik Freiburg gespeichert (siehe Datenschutzkonzept).

5. Wie werden die Datenspender angemessen und verständlich über die Nutzung ihrer Daten informiert?
   In Informationsschrift und Einwilligungserklärung (siehe Datenschutzkonzept).

6. Findet eine Weiterleitung von Projektdaten an Dritte oder ins Ausland statt?
   Nein – in der Nutzung der ChatGPT Modelle sind keine Daten betroffen, es werden nur methodische Fragen eingereicht.

7. Ist die Herkunft gekaufter Datensätze geklärt und sind diese Datensätze unter forschungsethisch vertretbaren Bedingungen gewonnen worden?

   Keine gekauften Datensätze.

8. Sind personenbezogene (auch pseudonymisierte) Daten ausreichend geschützt, und ist eine Re-Identifizierung anonymisierter Daten ausgeschlossen?
   Es werden keine personenbezogenen oder schützenswerten Daten in ChatGPT verarbeitet. Weitere Details im Datenschutzkonzept.

9. Mit welchen Daten wird/wurde das KI-System trainiert und können diese für das aktuelle Projekt als repräsentativ angesehen werden?

   ChatGPT nutzt als freie Basis große Teile der offenen wissenschaftlichen Literatur. Die vom Modell erzeugten Bausteine können für einige Fragen der statistischen Beratung als zutreffend erkannt werden. Detailliertere Erfahrungen und Rückmeldungen werden im Projekt EXPOLS erzeugt.

10. Im Fall von proprietärer Software und falls die Trainingsdaten und deren Repräsentativität nicht bekannt sind, geben Sie bitte den von den Vertreibern vorgesehenen Einsatzbereich an.
Allgemeingültiges Modell, welches aber mit dem Modul „Advanced Data Analyses“ auf Datenauswertung spezifiziert wurde. Dieses Modul wird hier aber nur für die Beantwortung von methodischen Fragen benutzt.

11. Sind inhärente Risiken des Systems für Verzerrungen, Reproduzierbarkeit, falsch positive und falsch negative Ergebnisse bekannt? Wurde das System auf solche Risiken getestet?

    Im Projekt EXPOLS wird die KI als Werkzeug benutzt. Die Beratung findet aber immer mit einem menschlichen Experten statt, der die Rückmeldungen kritisch hinterfragt und dem Beratenen dies spiegelt. Mögliche Verzerrungen durch Fehler werden also durch menschliche Expertise ausgeglichen.

12. Kann das Ergebnis des Projektes zu Diskriminierungseffekten führen (Geschlecht, Ethnie, Behinderte, Altersgruppen, …)?

    Nein

13. Sind im Projekt Tests, Methoden oder Screenings irgendeiner Art vorgesehen, mit deren Hilfe Verzerrungseffekte oder Diskriminierungseffekte aufgedeckt werden können?
Durch Beteiligung und Bewertung eines menschlichen Experten in der Beratung sollen alle derartigen Effekte aufgedeckt werden können.

14. Wo werden Daten, System und Algorithmen nachhaltig und zur Weiterverwendung dokumentiert?
Hier nicht relevant

15. Wurde das System als Medizinprodukt zertifiziert oder soll dies im Rahmen dieses Projektes oder später geschehen. Wenn ja, für welche Fragestellung (z.B. Selbstdiagnose App)?

    Nein
