# Supplementary material for: Exploring the potential of large language models for integration into an academic statistical consulting service–the EXPOLS study protocol
Source: PLoS One. 2024 Dec 4;19(12):e0308375. doi: 10.1371/journal.pone.0308375 (PMC11616834; doi:10.1371/journal.pone.0308375)
Supplement: Attachment — Submitted filename: Commented.docx [file pone.0308375.s003.docx]

**Major Points of Review**
1. The title is interesting and ideal, make it summarize the specific

**Part 1: Introduction**

1. The introduction does not clearly articulate the problems of data analysis or the rationale for this specific study regarding the Potential of Large language models for integration into an academic Statistical Consulting Service. For example, it should explain the problem of data quality, the problem of statistical methods, and the gap of statistical software or another specific purpose. The novelty of large language models should be articulated, highlighting any advancements.

**Part 2: The integration of an LLM into statistical consulting.**

1. The integration of LLM into statistical consulting is not well explained for the **pre-session preparation, data analysis guidance, and real-time assistance as well as assessing integration.**
2. Some of the research questions are not measurable. For instance, how to measure, efficiency, utility, and user satisfaction?

**Study Design**

1. Why incorporate qualitative methods**,** b/c more of such types of scenarios are more appropriate with quantitative methods if it is possible, please clearly articulate the reason for why.
2. The qualitative and quantitative methods are different aspects, so how do design at a time?
3. Is the given study population representative?

**Part 3: Statistical methods**

1. Qualitative and quantitative analysis is much more limited, regarding data type, purpose focuses, and others will not provide.

**Part 4: Novelty and Contribution**

The paper claims to contribute to improving the LLM. The novelty of this contribution is not fully established. The manuscript should better situate its work within the existing literature on LLM, explaining how it advances the state-of-the-art.

**Minor Points of Review:**

1. Clarity and Readability: the manuscript contains some typographical errors that need corrections for better readability. The structure of the manuscript could be improved by clearly delineating the sections on introductions, methods, and discussions.

Subheadings and bullet points can help in organizing the content more logically.

**Conclusion:** The study addresses an important issue for LLMs in supporting statistical consulting. Therefore, the manuscript requires substantial revisions. Furthermore, the novelty and contribution of the work are not established. Addressing the points raised above will be essential for improving the quality and impact of the manuscript.
